# A routine biomarker-based risk prediction model for metabolic syndrome in urban Han Chinese population

**DOI:** 10.1186/s12889-015-1424-z

**Published:** 2015-01-31

**Authors:** Wenchao Zhang, Qicai Chen, Zhongshang Yuan, Jing Liu, Zhaohui Du, Fang Tang, Hongying Jia, Fuzhong Xue, Chengqi Zhang

**Affiliations:** Department of Epidemiology and Biostatistics, School of Public Health, Shandong University, Jinan, 250012 China; Shengli Qilfield Central Hospital, Dongying, 257034 China; Health Management Center, Shandong Provincial QianFoShan Hospital, Jinan, 250014 China; The Second Hospital of Shandong University, Jinan, 250033 China

**Keywords:** Metabolic Syndrome (MetS), Routine biomarkers, Predictor model, Risk matrix

## Abstract

**Background:**

Many MetS related biomarkers had been discovered, which provided the possibility for building the MetS prediction model. In this paper we aimed to develop a novel routine biomarker-based risk prediction model for MetS in urban Han Chinese population.

**Methods:**

Exploring Factor analysis (EFA) was firstly conducted in MetS positive 13,345 males and 3,212 females respectively for extracting synthetic latent predictors (SLPs) from 11 routine biomarkers. Then, depending on the cohort with 5 years follow-up in 1,565 subjects (male 1,020 and female 545), a Cox model for predicting 5 years MetS was built by using SLPs as predictor; Area under the ROC curves (AUC) with 10 fold cross validation was used to evaluate its power. Absolute risk (AR) and relative absolute risk (RAR) were calculated to develop a risk matrix for visualization of risk assessment.

**Results:**

Six SLPs were extracted by EFA from 11 routine health check-up biomarkers. Each of them reflected the specific pathogenesis of MetS, with inflammatory factor (IF) contributed by WBC & LC & NGC, erythrocyte parameter factor (EPF) by Hb & HCT, blood pressure factor (BPF) by SBP & DBP, lipid metabolism factor (LMF) by TG & HDL-C, obesity condition factor (OCF) by BMI, and glucose metabolism factor (GMF) by FBG with the total contribution of 81.55% and 79.65% for males and females respectively. The proposed metabolic syndrome synthetic predictor (*MSP*) based predict model demonstrated good performance for predicting 5 years MetS with the AUC of 0.802 (95% CI 0.776-0.826) in males and 0.902 (95% CI 0.874-0.925) in females respectively, even after 10 fold cross validation, AUC was still enough high with 0.796 (95% CI 0.770-0.821) in males and 0.897 (95% CI 0.868-0.921) in females. More importantly, the *MSP* based risk matrix with a series of risk warning index provided a feasible and practical tool for visualization of risk assessment in the prediction of MetS.

**Conclusions:**

MetS could be explained by six SLPs in Chinese urban Han population. The proposed *MSP* based predict model demonstrated good performance for predicting 5 years MetS, and the MetS-based matrix provided a feasible and practical tool.

**Electronic supplementary material:**

The online version of this article (doi:10.1186/s12889-015-1424-z) contains supplementary material, which is available to authorized users.

## Background

Metabolic syndrome (MetS) is a disorder with co-occurrence of several known cardiovascular risk factors, including insulin resistance, obesity, atherogenic dyslipidemia and hypertension [1]. With the economic development and the changing of people's lifestyle in china, the prevalence of MetS is increasing rapidly. Compared with Europeans and Americans, Asians are more likely to have MetS [2]. Data from the China Health and Nutrition Survey conducted in 2009 suggested that the prevalence rate of MetS has reached up to 21.3% among the Chinese adults [3]. Many studies indicated that incidence of MetS will increase the risk of type 2 diabetes [4], cardiovascular disease [5-9], renal damage [10,11], and so on. Therefore, prediction of MetS is very essential for early prevention of the above diseases.

Some risk scores based on cross-sectional studies were structured for screening undiagnosed MetS [12-14], which depended on questionnaire survey about participants’ lifestyle and medical histories. Although the area under the ROC curves for detecting the MetS in these studies were acceptable with a range from 72.4% to 80.1%, cross-sectional study could only provide temporal information of the subjects. Cohort study is more preferable for risk assessment. Hsiao and Yang conducted a two-year (from 2003 to 2005) [15] and a 5-year follow-up study (during 1997–2006) [16] respectively in Chinese population. Both of them confirmed that routine check-up biomarkers like serum cholesterol, triglycerides, blood glucose, measurement of body height and weight, blood pressure et al., could be served as effective predictors to MetS using multivariate logistic regression (MLR). However, MLR is not suitable for survival data, and it also limited the applying of the model in the first study due to relative short follow-up time and small sample. In the second study stepwise regression has ruled out many MetS related biomarkers from the model. Fortunately, many other studies [17-29] have found a number of MetS related biomarkers, which provide us a convenience to build the risk appraisal model of MetS. After studying 6Synthetic Latent Predictors from 11 MetS routine biomarkers in a MetS positive population, we develop a novel routine biomarker-based risk prediction model for MetS in urban Han Chinese population.

## Methods

### Participants

Subjects were selected from the urban adult citizen who came to the Center for Health Management of Shandong Provincial QianFoShan Hospital, and the Health Examination Center of Shandong Provincial Hospital to conduct medical examination from 2005 to 2010. Among 92,284 subjects (aged 18 to 82 years) completing all steps of physical examination, 16,557 subjects were diagnosed with MetS at their first check-up year according to the criteria of the Chinese Medical Association. Of 75,727 subjects without MetS at baseline, 1,565 (1,020 males and 545 females) completed a 5-year follow-up and were included in the cohort study design. The cumulative incidence rate was calculated for 1,565 subjects who were followed up.

### Biomarkers selection and measurements

In the present study, eleven biomarkers were selected from routine health check-up data, including body mass index (BMI), systolic blood pressure (SBP), diastolic blood pressure (DBP), fasting blood-glucose (FBG), triglycerides (TG), high-density lipoprotein cholesterol (HDL-C), hemoglobin (Hg), hematocrit (HCT), white blood cell count (WBC), lymphocyte (LC), neutrophile granulocyte (NGC). Among them, BMI, SBP, DBP, TG, HDL-C and FBG were selected based on the traditional definition of MetS. The others were included according to the available results of peer studies: Hb [17-19], HCT [17,18,28], WBC [20-27], LC [23,29], NGC [23,29]. All measurements were conducted in the Center for Health Management of Shandong Provincial QianFoShan Hospital and the Health Examination Center of Shandong Provincial Hospital following same and standard procedures. Both of the two institutions are nationally accredited. The whole study was approved by the Ethics Committee of School of Public Health, Shandong University, and written informed consent was obtained from all eligible participants.

### Definition of MetS

Chinese Medical Association Diabetes Branch criteria [30] were applied to define MetS in this paper. Subjects who had three or more of the following four signs were diagnosed with MetS: 1) overweight or obesity, BMI ≥25.0 Kg/M^2^; 2) hypertension, systolic/ diastolic ≥140 mmHg/90 mmHg or previous diagnosis; 3) dyslipidemia, fasting TG ≥1.7 mmol/L (110 mg/dl), or fasting high-density lipoprotein cholesterol (HDL-C) <0.9 mmol/L (35 mg/dl); 4) hyperglycemia, fasting blood-glucose (FBG) ≥6.1 mmol/L(110 mg/dl) or 2 h Post-meal glucose (PG) ≥7.8 mmol/L(140 mg/dl), or previous diagnosis.

### Statistical analysis

Descriptive statistics were conducted for 16,557 subjects with MetS at baseline. Student's *t* test was used to detect the statistical significances for 11 biomarkers between males and females, and the χ^2^ test was conducted to detect the difference in the prevalence of the four basic components (obesity, hypertension, dyslipidemia and hyperglycemia) between males and females.

To eliminate multicollinearity between the routine check-up biomarkers and build a better model for MetS prediction, both Exploring factor analysis(EFA) and Cox proportional hazard regression model are applied in the present study. Finally, a MetS synthetic predictor (*MSP*) was developed through the following four steps: First, EFA with principal component algorithm and varimax rotation from correlation matrix was performed to extract independent MetS risk-related factors from the 11 routine check-up biomarkers in 16,557 subjects with MetS at baseline. The criteria for retaining factors in the present study was eigenvalue >0.9 (for keeping the accounting variations of total was greater than 70%). Only variables that share a factor loading of at least 0.50 were used for further analytical interpretation and named factors. Second, a Cox regression model was built between the hazard function of MetS and the extracted factors in 1,565 subjects in the cohort study design: h(t) = h0(t)exp(β_0_age + β_1_F_1_ + β_2_F_2_ + … + β_k_F_k_), and a *MSP* was developed by *MSP* = β_1_F_1_ + β_2_F_2_ + … + β_k_F_k_. Third, the risk of MetS, for the 1565 subjects from the cohort, was estimated by

1 $$ P(t)=1-S(t)=1- \exp \left(-{\displaystyle {\int}_0^t{h}_0(u) \exp (B)du}\right) $$

where, *P(t)* was the predictive probability of MetS at year *t*, *B* = *β*_0_*age* + *β*_1_*MSP*. For both training set and 10 fold cross validation, Receiver Operator Characteristics curve (ROC curve) analysis was conducted, and the area under the ROC curve (AUC) together with sensitivity, specificity, 95% Confidence Interval and cut-off of *P* value was calculated by MedCalc software [31]. The optimal cut-off was estimated based on the Youden index criterion [32] which is optimal in the sense that it provides a score which reflects the intention of maximizing the overall correct classification rate. Finally, Excess Absolute Risk (EAR) and Relative Absolute Risk(RAR) were calculated for 1,565 subjects from the five-year follow-up who had completed physical examinations and the 11 biomarker measurements by $EAR={P}_j(t)-{\overline{P}}_j(t)$ and $RAR={P}_j(t)/{\overline{P}}_j(t)$ respectively, where *P*_*j*_(*t*) signified Absolute Risk (AR), namely the probability of MetS at year *t*, in which *j* noted subject’s age. ${\overline{P}}_j(t)$ signified the average probability of MetS at year *t* in *j*^*th*^ age, which can be calculated by model (1) through ${\overline{B}}_j={\beta}_1 ag{e}_j+\gamma {\overline{MSP}}_j$, where ${\overline{MSP}}_j$ was the mean of *MSP* in *j*^*th*^ age. All the steps were conducted in males and females respectively. The risk matrix for AR and RAR were depicted using ArcGIS 9.1, and all statistical analyses was performed using SAS 9.1.3 with *P* < 0.05 considered statistically significant.

## Results

The prevalence of MetS in the study was 17.9% (16,557/92,284) (22.7% in males and 9.6% in females) at baseline. At the end of the follow-up period of 1,565 subjects, 348 incident MetS cases (286 males and 62 females) were diagnosed and the cumulative incidence rate was 22.2% (28% in males and 11.4% in females) (see Additional file 1: Table S1). The prevalence of four basic components (obesity, hypertension, hyperglycemia, and dyslipidemia) was significantly different between males and females (see Additional file 2: Table S2).

Table 1 showed the distribution of age and eleven biomarkers between males and females with MetS at baseline, indicating that all variables except BMI and LC were significantly different between males and females. Of them, DBP, TG, Hb, HCT, WBC and NGC were higher in males than in females, while age, SBP, FBG, and HDL-C were higher in females than in males. Correlation matrix between 11 biomarkers was illustrated in Additional file 3: Table S3. The results of EFA were showed in Table 2 with explained variance and cumulative variance, this suggested that six synthetic latent predictors (SLPs) could explain 81.55% and 79.65% of total variance for males and females respectively. According to the criteria of analytical interpretation stated in the statistical analysis section, they were named as inflammatory factor (IF), erythrocyte parameter factor (EPF), blood pressure factor (BPF), lipid metabolism factor (LMF), obesity condition factor (OCF), and glucose metabolism factor (GMF) in both males and females. Of the six SLPs, IF was contributed by WBC & LC & NGC, EPF by Hb & HCT, BPF by SBP & DBP, LMF by TG & HDL-C, OCF by BMI, and GMF by FBG.

**Table 1:** **Distribution of age and the eleven biomarkers between male and female with baseline metabolic syndrome**

	**Male (n = 13345)**	**Female (n = 3212)**	**P values**
	**Mean ± SD**	**Mean ± SD**	
**Age (years)**	49.00 ± 13.09	59.50 ± 12.49	<.0001
**body mass index (kg/m** ^**2**^ **)**	28.29 ± 2.99	28.27 ± 3.10	0.8241
**systolic blood pressure (mmHg)**	144.60 ± 17.28	150.40 ± 20.54	<.0001
**diastolic blood pressure (mmHg)**	87.09 ± 12.00	81.76 ± 11.99	<.0001
**fasting blood-glucose (mmol/L)**	6.43 ± 1.94	6.65 ± 2.09	<.0001
**triglycerides (mmol/L)**	2.87 ± 2.26	2.42 ± 1.53	<.0001
**high-density lipoprotein cholesterol (mmol/L)**	1.18 ± 0.35	1.32 ± 0.35	<.0001
**hemoglobin (g/L)**	157.70 ± 10.82	138.30 ± 11.88	<.0001
**hematocrit (%)**	46.27 ± 3.03	41.65 ± 3.18	<.0001
**white blood cell count (10** ^**9**^ **/L)**	7.18 ± 1.67	6.90 ± 1.63	<.0001
**lymphocyte (10** ^**9**^ **/L)**	2.25 ± 0.69	2.24 ± 0.68	0.4432
**neutrophile granulocyte (10** ^**9**^ **/L)**	4.30 ± 1.28	4.13 ± 1.27	<.0001

**Table 2:** **Factor loadings by principal component analysis with varimax rotation on 11 routine health check-up biomarkers in MetS patients**

	**Males (n = 13345)**						**Females (n = 3212)**					
	**IF**	**EPF**	**BPF**	**LMF**	**OCF**	**GMF**	**IF**	**EPF**	**BPF**	**LMF**	**OCF**	**GMF**
**BMI**	0.050	0.017	0.108	−0.042	**0.954**	0.007	0.016	0.001	0.184	0.080	**0.873**	−0.013
**SBP**	0.031	−0.088	**0.885**	−0.051	−0.007	0.039	0.018	−0.019	**0.851**	0.013	0.015	0.023
**DBP**	0.001	0.206	**0.813**	0.122	0.134	−0.101	0.005	0.155	**0.786**	−0.012	0.120	−0.065
**FBG**	−0.001	−0.017	−0.049	0.111	0.008	**0.965**	0.074	0.038	−0.041	0.165	0.004	**0.905**
**TG**	0.066	0.051	−0.032	**0.825**	0.171	0.087	0.013	0.067	−0.045	**0.776**	0.082	0.199
**HDL-C**	−0.050	−0.043	0.093	**0.794**	−0.239	0.027	−0.076	−0.039	0.131	**0.583**	−0.546	−0.027
**Hb**	0.045	**0.971**	0.051	0.021	0.010	0.018	0.047	**0.975**	0.071	0.028	0.009	0.055
**HCT**	0.119	**0.957**	0.041	−0.012	0.017	−0.040	0.095	**0.969**	0.074	0.045	0.013	−0.017
**WBC**	**0.991**	0.078	0.035	−0.020	0.010	0.036	**0.992**	0.076	0.014	0.045	0.033	0.013
**LC**	**0.642**	0.033	−0.072	0.127	0.117	−0.176	**0.592**	0.086	−0.031	0.410	0.109	−0.317
**NGC**	**0.853**	0.073	0.086	−0.094	−0.058	0.144	**0.881**	0.040	0.033	−0.173	−0.025	0.197
**% Variance explained**	22.15	15.87	13.34	12.93	8.88	8.37	22.21	16.21	13.15	11.12	9.01	7.95
**Cumulative variance**	22.15	38.03	51.37	64.30	73.18	81.55	22.21	38.42	51.57	62.69	71.70	79.65

BMI indicated body mass index; SBP, systolic blood pressure; DBP, diastolic blood pressure; FBG, fasting blood-glucose; TG, triglycerides; HDL-C, high-density lipoprotein cholesterol; Hb, hemoglobin; HCT, hematocrit; WBC, white blood cell count; LC, lymphocyte; NGC, neutrophile granulocyte; Factor1-Factor6 was called as inflammatory factor (IF), erythrocyte parameter factor (EPF), blood pressure factor (BPF), lipid metabolism factor (LMF), obesity condition factor (OCF), glucose metabolism factor (GMF). The bold character figures were factor loadings greater than 0.50.

**Figure 1:**
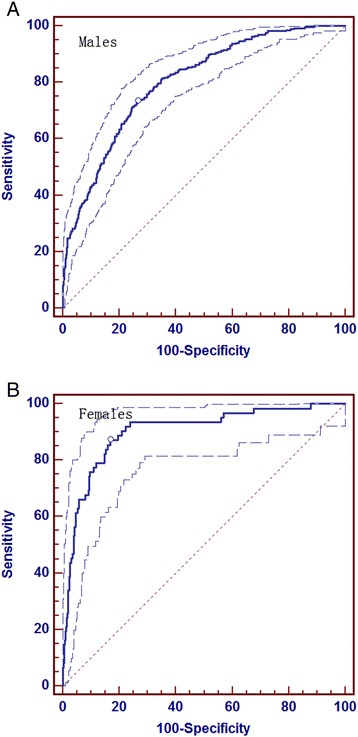
**ROC curve for prediction of metabolic syndrome.** A shows the predictive effect in males, B represents corresponding result in females. The dotted plots stand for 95% Confidence Interval. **A**: Area under the ROC curve (AUC) 0.802; Standard Error 0.0168; 95% Confidence Interval 0.776 to 0.826; z statistic 17.916; Significance level P (Area = 0.5) 0.0001. Point with the highest accuracy showed sensitivity 73.4 and specificity 73.3 under the cut-off 0.2749. **B**: Area under the ROC curve (AUC) 0.902; Standard Error 0.0264; 95% Confidence Interval 0.874 to 0.925; z statistic 15.233; Significance level P (Area = 0.5) 0.0001. Point with the highest accuracy showed sensitivity 87.1 and specificity 83.0 under the cut-off 0.1181.

Figure 1 showed the result of ROC analysis to predict 5-year risk of MetS by the proposed predict model. It indicated that the AUC was up to 80.2% and 90.2% for males and females respectively in training set (seeing Figure 1A and 1B). While 79.6% and 89.7% after 10 fold cross validation.

**Figure 2:**
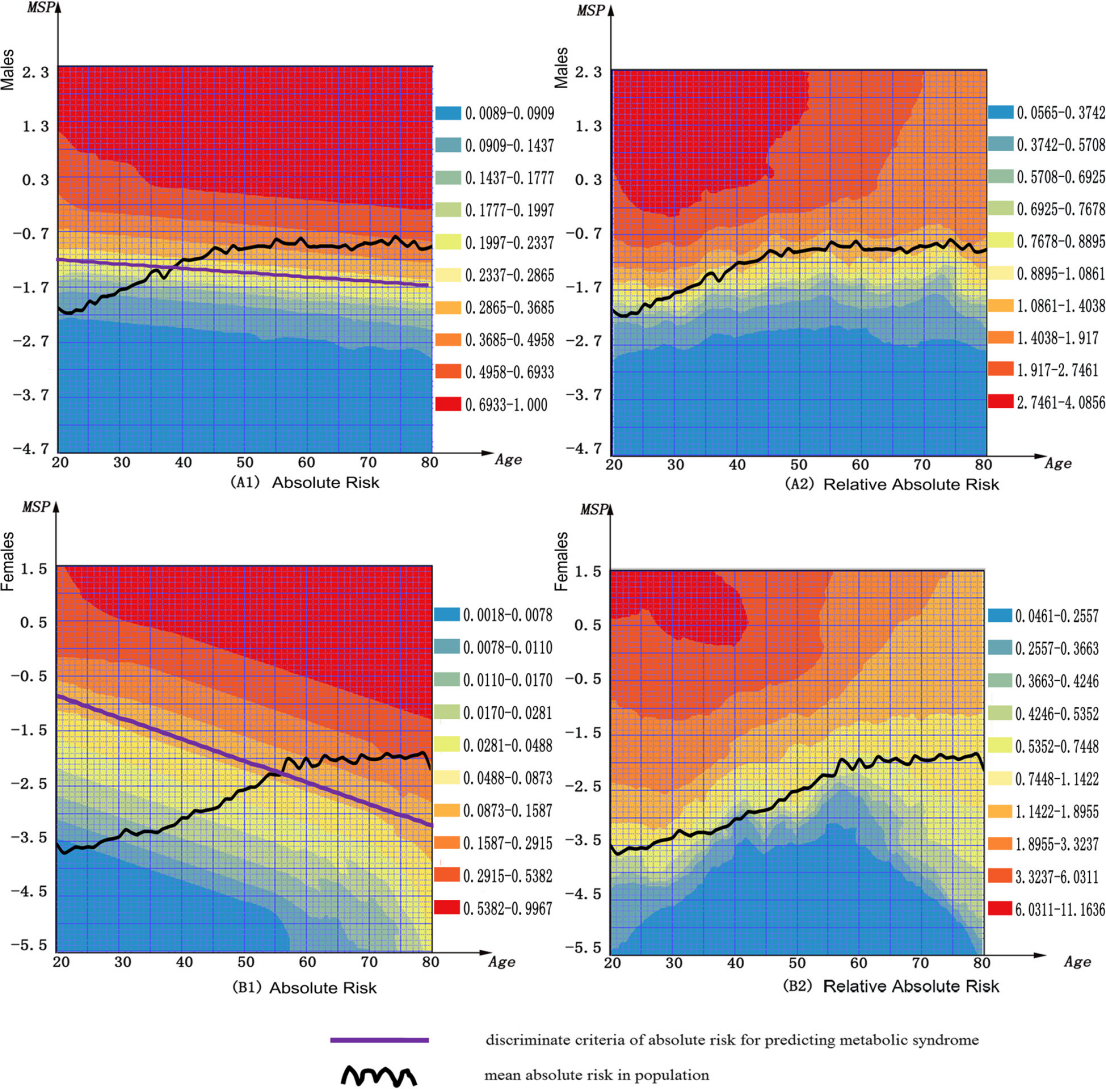
**The 5-year risk matrix for risk appraisal of metabolic syndrome by gender.** **A1** and **B1** are absolute risk matrix of male and female respectively. **A2** and **B2** are relative absolute risk matrix of male and female respectively. For male: MetS Predictor (*MSP*) = 0.451604BMI + 0.313187SBP + 0.250746DBP +0.670039 FB G + 0.120262TG-0.06067HLD_C + 0.042693Hb + 0.003179HCT + 0.064581WBC-0.08385LC + 0.126292NGC. For female: MetS Predictor (*MSP*) = 0.711655BMI + 0.266298SBP + 0.290385DBP + 0.392424FBG + 0.482012TG-0.09606HLD_C + 0.116441Hb + 0.10335HCT + 0.07158WBC + 0.160117LC-0.0048NGC.

Figure 2 showed the 5-year AR matrix and RAR matrix for MetS by gender in the cohort (n = 1,565), specifically Figure 2A1 and 2A2 for males, and Figure 2B1 and 2B2 for females. These matrices provide a convenient tool for conducting MetS prediction in health management and clinical practice. For example, a man aged 30-year-old and having AR of 0.233 has an EAR of 0.054 (0.233-0.179) and RAR of 1.301 (0.233/0.179), while a man aged 60-year-old and having an AR of 0.233, has the EAR −0.187 (0.233-0.420) and RAR of 0.555 (0.233/0.420). These show that although their predictive probabilities for MetS over 5 years are the same, the younger man has a higher MetS risk compared to his peers, about 1.301 times than that of the average risk of 40-year-old population, indicating that changes in lifestyles and social intervention strategies are needed for him. Alternatively the MetS risk of the older man is lower than the average risk of the same age, only 55.5% of the average risk of 60-year-old population, indicating that he has a good health status compared with his peers.

**Figure 3:**
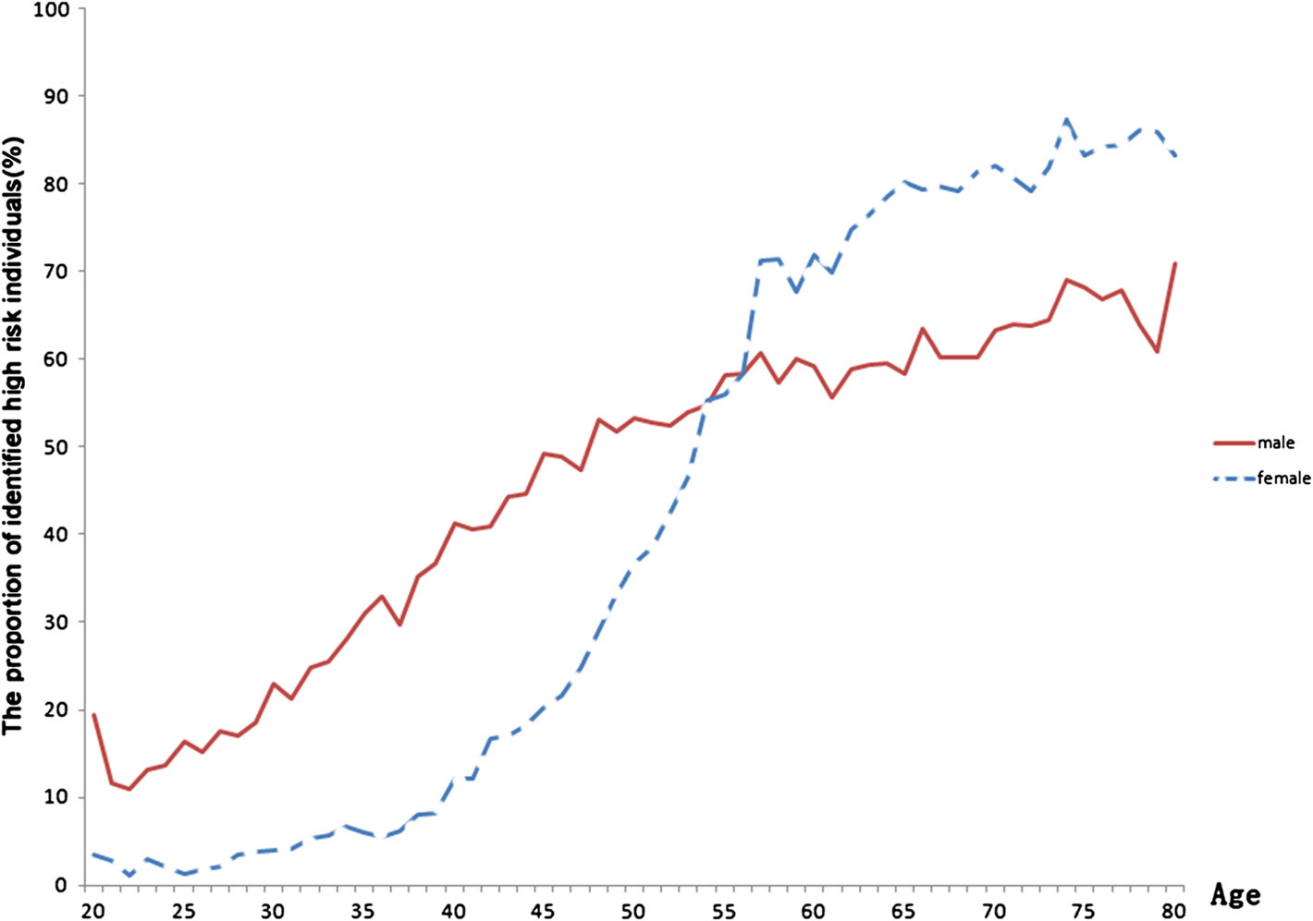
**The metabolic syndrome risk appraisal result of 92284 subjects in routine health check-up system.**

Using the cut-off points showed in Figure 1A for males (0.2749) and Figure 1B for females (0.1181), people were classified as high-risk population (> the cut-off point value) or low-risk population (≤ the cut-off point value). The proportion of high-risk that comes with ageing in the general population (n = 92284) was drawn in Figure 3. Generally, the proportion of high risk subjects increase with age in both males and females. Nevertheless, the proportion of high-risk was higher in males than females before the age of 55, while it was the reverse after 55.

## Discussions

### The routine health check-up based biomarkers for predicting MetS

Currently, several potential routine health check-up based biomarkers, such as Hb [17-19], HCT [17,18,28], WBC [20-27], LC [23,29] and NGC [23,29], were identified for predicting MetS/its components. Correlation matrix between 11 biomarkers was illustrated in Additional file 3: Table S3, which Shows the necessity of EFA. In this paper, we extracted 6 independent synthetic latent predictors (SLPs) by EFA from 11 routine health check-up biomarkers (BMI, SBP, DBP, FBG, TG, HDL-C, Hb, HCT, WBC, LC, NGC), not only with their specific clinical significances, but eliminating the multicollinearity between them. Each SLPs reflected the specific pathogenesis of MetS, with IF contributed by WBC & LC & NGC, EPF by Hb & HCT, BPF by SBP & DBP, LMF by TG & HDL-C, OCF by BMI, and GMF by GMF (see Table 2). The cumulative Variances explained by the six SLPs were up to 81.55% and 79.65% for males and females respectively. Particularly, the IF and EPF were identified as the key factors for the variation of MetS with their contribution proportion of 22.25% &15.87% in males and 22.21%&16.21% in females respectively. Pathogenically, both of them were strong associated with insulin resistance [33-36] which was the ‘core’ for MetS [37-39]. EPF was contributed by Hb & HCT. Hb is a carrier and buffer of nitric oxide (NO), and various compounds of Hb with NO can affect Hb-oxygen affinity of the whole blood [40]. Disturbed NO synthesis may exert an adverse effect on endothelial dysfunction through the L-arginine-NO pathway [41]. Furthermore, endothelial dysfunction was reported to be associated with MetS [42,43]. HCT could change blood viscosity and peripheral resistance to blood flow, and further contribute to insulin resistance [44-46].

### Metabolic syndrome synthetic predictor and its application in MetS prediction

At the end of the follow-up period, the cumulative incidence rate reach up to 22.2% (28% in males and 11.4% in females) (see Additional file 1: Table S1). Currently, three cross-sectional design based risk scores [12-14] and two cohort design based predictive models [15,16] had been developed to predict MetS on different ethnicities. Although these predict tools obtain acceptable power with their AUC ranged from 0.724 to 0.827, their risk algorithm and visualization of risk assessment still had development potential for improving power, feasibility and practicability. In this paper we developed a routine health check-up cohort design based MetS synthetic predictor (*MSP*) for predict 5 year risk of MetS in the frame work of Cox regression model. The *MSP* based predict model demonstrated good performance for predicting 5 years MetS with the AUC of 0.802 (95% CI 0.776-0.826) in males and 0.902 (95% CI 0.874-0.925) in females respectively, even after 10 fold cross validation, AUC was still enough high with 0.796 (95% CI 0.770-0.821) in males and 0.897 (95% CI 0.868-0.921) in females. More importantly, the *MSP* was further used to construct the risk matrix with a series of risk warning indexes including average risk in population, AR & RAR for subjects, and the cut-off curve for predicted MetS (see Figure 2). This matrix provided a feasible and practical tool for visualization of risk assessment in the prediction of MetS. As an example, for a woman at a given age who receives health check-up, the risk matrices can provide her with AR (Figure 2B1) and RAR (Figure 2B2) compared with the average hazard within the same age group in females, this may urge her to intervene risk factors for reducing risk of MetS.

### The risk distribution in urban Han Chinese population

The proportion of subjects with high-risk was higher in males than females before the age of 55, while it was in reverse after 55 (showed in Figure 3). Similar results have been obtained in the Korean population [47] with the demarcation point of 60 years old. In particular, the patterns of subjects with high-risk were quite different between males and females. The proportion of subjects with high-risk increased linearly with age in male population, while showed an S shaped curve in female population with the fastest growth period from 40 to 60 years old. This difference may be associated with women’s menopause. Various studies indicated that natural menopause was associated with increased central adiposity [48], blood pressure [49-55], total cholesterol, LDL cholesterol and triglyceride levels [50,56], which would further increase risk of MetS during the menopause transition years. The contribution of several metabolic components to the metabolic syndrome is different in males and females (see Additional file 2: Table S2).

We re-assessed the predictive ability using IF & EPF alone and four classical MetS components respectively. Additional file 4: Table S4 showed these results, as expected, our proposed 6 SLPs still have the best performance. Actually, in China, health check up was embedded in “physical examination package” and usually the biomarkers can be obtained together. The Chinese government request every Health Examination Center that at least all biomarkers used in this manuscript must be tested during the health examination process.

### Limitations

Study population was just employed urban residents, therefore the results may not extend to general population. In addition, five years follow-up was relatively short for predict long term risk of MetS.

## Conclusions

In conclusion, MetS could be explained by six SLPs in Chinese urban Han population. The proposed *MSP* based predict model demonstrated good performance for predicting 5 years MetS, and the MetS-based matrix provided a feasible and practical tool for visualization of risk assessment in the prediction of MetS.

## Additional files

Additional file 1: Table S1. The incidence rate of MetS in unaffected 1,565 subjects at baseline after a follow-up of five years. Additional file 2: Table S2. The prevalence of the 4 basic components for both male and female metabolic syndrome groups. Additional file 3: Table S3. Correlation matrix between eleven biomarkers. Additional file 4: Table S4. The area under the ROC curves (AUC) by using different MetS predictors.
