# Supplementary material for: A routine biomarker-based risk prediction model for metabolic syndrome in urban Han Chinese population
Source: BMC Public Health. 2015 Jan 31;15:64. doi: 10.1186/s12889-015-1424-z (PMC4320489; doi:10.1186/s12889-015-1424-z)
Supplement: Additional file 1: Table S1. — The incidence rate of MetS in unaffected 1,565 subjects at baseline after a follow-up of five years. [file 12889_2015_1424_MOESM1_ESM.doc]

**Table S1 The incidence rate of MetS in unaffected 1565 subjects at baseline after a follow-up of five years**

| **Gender** | **2005** | **2006** | **2007** | **2008** | **2009** | **2010** | **cumulative incidence rate (%)** |
| --- | --- | --- | --- | --- | --- | --- | --- |
| **Male (n=1020)** | 0 | 59 | 61 | 63 | 47 | 56 | 28 |
| **Female (n=545)** | 0 | 16 | 18 | 12 | 7 | 9 | 11.4 |
