# Supplementary material for: A routine biomarker-based risk prediction model for metabolic syndrome in urban Han Chinese population
Source: BMC Public Health. 2015 Jan 31;15:64. doi: 10.1186/s12889-015-1424-z (PMC4320489; doi:10.1186/s12889-015-1424-z)
Supplement: Additional file 2: Table S2. — The prevalence of the 4 basic components for both male and female metabolic syndrome groups. [file 12889_2015_1424_MOESM2_ESM.doc]

**Table S2 The prevalence of the 4 basic components for both male and female metabolic syndrome groups.**

| **Component** | **Male(n=13,345)** | | **Female(n=3,212)** | |
| --- | --- | --- | --- | --- |
|  | **n** | **%** | **n** | **%** |
| **Obesity** | 12850 | 96.29 | 2994 | 93.21 |
| **Hypertension** | 11063 | 82.90 | 2796 | 87.05 |
| **Hyperglycemia** | 6878 | 51.54 | 1861 | 57.94 |
| **Dyslipidemia** | 12164 | 91.15 | 2675 | 83.28 |

All p values were <0.0001.
