# Supplementary material for: A routine biomarker-based risk prediction model for metabolic syndrome in urban Han Chinese population
Source: BMC Public Health. 2015 Jan 31;15:64. doi: 10.1186/s12889-015-1424-z (PMC4320489; doi:10.1186/s12889-015-1424-z)
Supplement: Additional file 3: Table S3. — Correlation matrix between eleven biomarkers. [file 12889_2015_1424_MOESM3_ESM.doc]

**Table S3 Correlation matrix between eleven biomarkers.**

|  | **BMI** | **SBP** | **DBP** | **FBG** | **TG** | **HDL_C** | **Hb** | **HCT** | **WBC** | **LC** | **NGC** |
| --- | --- | --- | --- | --- | --- | --- | --- | --- | --- | --- | --- |
| **BMI** | 1 | 0.324833* | 0.298769* | 0.106884* | 0.2322* | -0.27004* | 0.317449* | 0.304254* | 0.184597* | 0.145548* | 0.141782* |
| **SBP** |  | 1 | 0.622357* | 0.176467* | 0.101768* | -0.04266 | 0.19495* | 0.195489* | 0.063606 | 0.063787* | 0.043994 |
| **DBP** |  |  | 1 | 0.098409* | 0.170132* | -0.04675 | 0.285137* | 0.291173* | 0.130364* | 0.09745* | 0.102146* |
| **FBG** |  |  |  | 1 | 0.111942* | 0.002206 | 0.013832* | -0.00732 | -0.01875 | 0.129339* | -0.08363* |
| **TG** |  |  |  |  | 1 | -0.06219* | 0.246274* | 0.229284* | 0.16603* | 0.146581* | 0.120869* |
| **HDL-C** |  |  |  |  |  | 1 | -0.29489* | -0.28956* | -0.1832* | -0.08163* | -0.16876* |
| **Hb** |  |  |  |  |  |  | 1 | 0.964539* | 0.236526* | 0.118215* | 0.210476* |
| **HCT** |  |  |  |  |  |  |  | 1 | 0.258305* | 0.108713* | 0.239271* |
| **WBC** |  |  |  |  |  |  |  |  | 1 | 0.560673* | 0.911085* |
| **LC** |  |  |  |  |  |  |  |  |  | 1 | 0.188673 |
| **NGC** |  |  |  |  |  |  |  |  |  |  | 1 |

* P≤0.05
