# Supplementary material for: A routine biomarker-based risk prediction model for metabolic syndrome in urban Han Chinese population
Source: BMC Public Health. 2015 Jan 31;15:64. doi: 10.1186/s12889-015-1424-z (PMC4320489; doi:10.1186/s12889-015-1424-z)
Supplement: Additional file 4: Table S4. — The area under the ROC curves (AUC) by using different MetS predictors. [file 12889_2015_1424_MOESM4_ESM.doc]

**Table S4** The area under the ROC curves (AUC) by using different MetS predictors

|  | **IF &EPF** | **four components of MetS** | **6 SLPs** |
| --- | --- | --- | --- |
| **Male** | 54.4% | 73.5% | 80.2% |
| **Female** | 62.6% | 87.3% | 90.2% |
